# Size and sequence polymorphisms in the glutamate-rich protein gene of the human malaria parasite *Plasmodium falciparum* in Thailand

**DOI:** 10.1186/s13071-018-2630-1

**Published:** 2018-01-22

**Authors:** Sittiporn Pattaradilokrat, Chawinya Trakoolsoontorn, Phumin Simpalipan, Natapot Warrit, Morakot Kaewthamasorn, Pongchai Harnyuttanakorn

**Affiliations:** 1 Department of Biology, Faculty of Science, Chualongkorn University, 254 Phayathai Road, Bangkok, 10330 Thailand; 2 Veterinary Parasitology Research Group, Department of Pathology, Faculty of Veterinary Science, Chulalongkorn University, Bangkok, 10330 Thailand

**Keywords:** DNA sequence, Genetic variation, *Plasmodium falciparum*, Glutamate-rich protein

## Abstract

**Background:**

The glutamate-rich protein (GLURP) of the malaria parasite *Plasmodium falciparum* is a key surface antigen that serves as a component of a clinical vaccine. Moreover, the *GLURP* gene is also employed routinely as a genetic marker for malarial genotyping in epidemiological studies. While extensive size polymorphisms in *GLURP* are well recorded, the extent of the sequence diversity of this gene is rarely investigated. The present study aimed to explore the genetic diversity of *GLURP* in natural populations of *P. falciparum*.

**Results:**

The polymorphic C-terminal repetitive R2 region of *GLURP* sequences from 65 *P. falciparum* isolates in Thailand were generated and combined with the data from 103 worldwide isolates to generate a GLURP database. The collection was comprised of 168 alleles, encoding 105 unique GLURP subtypes, characterized by 18 types of amino acid repeat units (AAU). Of these, 28 GLURP subtypes, formed by 10 AAU types, were detected in *P. falciparum* in Thailand. Among them, 19 GLURP subtypes and 2 AAU types are described for the first time in the Thai parasite population. The AAU sequences were highly conserved, which is likely due to negative selection. Standard *Fst* analysis revealed the shared distributions of GLURP types among the *P. falciparum* populations, providing evidence of gene flow among the different demographic populations.

**Conclusions:**

Sequence diversity causing size variations in *GLURP* in Thai *P. falciparum* populations were detected, and caused by non-synonymous substitutions in repeat units and some insertion/deletion of aspartic acid or glutamic acid codons between repeat units. The *P. falciparum* population structure based on GLURP showed promising implications for the development of GLURP-based vaccines and for monitoring vaccine efficacy.

**Electronic supplementary material:**

The online version of this article (doi: 10.1186/s13071-018-2630-1) contains supplementary material, which is available to authorized users.

## Background

The rapid and accurate identification of malaria parasite species is critical for clinical malaria diagnosis and subsequent treatment. A microscopic examination of the blood stage malaria on blood films is the most commonly used method in the laboratory and clinics. This technique is simple, low-cost and easy to perform, but it has many limitations, such as the requirement of high parasitemia levels and skills for diagnostic interpretation. More importantly, microscopic examination alone cannot reveal the multiplicity of an infection, which is the feature that occurs in malaria-endemic regions. Molecular tools, such as the polymerase chain reaction (PCR), may be considered as a suitable alternative. A number of PCR-based diagnostic assays have been developed and increasingly adopted for clinical malaria diagnostics [1, 2]. For identification of malaria species, house-keeping genes have been chosen as molecular targets for developing PCR assays, including the small subunit ribosomal RNA gene in the nuclear genome as well as the cytochrome *c* oxidase subunit 1 gene in the mitochondrial genome [3–6]. Additionally, PCR-based assays can be performed using polymorphic loci, including amplified fragment length polymorphism, microsatellites and antigen-coding genes, facilitating the identification of the parasite strains, the identification of monoclonal vs polyclonal infections, and the differentiation between relapse vs re-infections [7–11]. One of the polymorphic antigen-encoding genes often employed as a key genetic marker for strain identification of the human malaria parasite *P. falciparum* is the glutamate-rich protein (*GLURP*) gene.

The glutamate-rich protein (GLURP), a 220 kDa antigen expressed throughout the life-cycle of the malaria parasites in a mammalian host [12, 13], is localized to the *Plasmodium falciparum* Pfs38 protein complex on the merozoite surface, and the protein contains a module of six cysteine residues forming three intramolecular disulphide bonds. The Pfs38 complex binds to host erythrocytes directly via glycophorin A as a receptor [14]. The GLURP protein consists of the three defined regions of an N-terminal non-repetitive region (R0), a central repetitive sequence (R1) and a C-terminal repetitive region (R2). The R0 region is predicted to be hydrophobic and may act as a signal peptide, which is a feature of secretory proteins [13].

Evidence that supports the role of GLURP as an immune target comes from several studies. First, immunizing mice with a vaccinia virus-expressed GLURP of *P. falciparum* induced a humoral immune response [15]. To further map the immunodominant region of GLURP, four purified polypeptides, including the full-length protein and the R0, R1 and R2 regions, were produced in *Escherichia coli* and then tested for reactivity with sera from adults living in endemic areas in Liberia. The antibody reactivity against the R2 and R0 regions was observed in nearly 100% of the serum samples, suggesting a role for the R2 and R0 regions as the immune target [16]. Subsequent epidemiological studies performed in endemic areas in Africa and South America consistently showed that malaria-infected patients had high levels of antibodies that react specifically with GLURP, and the levels of anti-GLURP antibodies against the R0 and R2 regions were positively associated with a reduced risk of clinical malaria [17–21]. Anti-GLURP antibodies against the R0 region act in synchronization with monocytes or mediate opsonic phagocytosis, conferring protection against febrile malaria [22, 23]. Together, these studies demonstrate that GLURP R0 and R2 may participate in eliciting protective immunity in humans, thereby giving further impetus for the development of malaria vaccines targeting GLURP.

Accordingly, *P. falciparum* GLURP has currently been employed in vaccine development. One example is the GMZ2 vaccine, which combines the immunodominant GLURP R0 region and MSP-3 of *P. falciparum*. The vaccine is designed to mimick naturally acquired anti-malarial immunity [24]. In pre-clinical studies, the vaccine appeared to be safe and induced strong protective immune responses against the malaria parasites in simian models [25, 26]. In Phase 1 clinical trials, GMZ2 was safe, well tolerated and immunogenic in both children and adults [27–29]. Serological analyses of the antibody levels showed high levels of specific and functional antibodies in vaccinated subjects, with the capacity to control the parasite multiplication [30, 31]. Following these trials, a Phase 2 clinical trial was conducted in participants of 1 to 5 years of age in four African countries, and the GMZ2 vaccine was confirmed to be well tolerated, immunogenic and capable of reducing the malaria incidence, but the efficacy was only moderate [32]. It is likely that the efficacy of the vaccine was compromised by the parasite antigenic diversity [33]. Therefore, it is necessary to investigate the extent of genetic polymorphism of any vaccine candidate for malaria parasites in the natural populations where the vaccine is to be used.

The *GLURP* gene of *P. falciparum* is located on chromosome 10 and encodes a long polypeptide of 1271 amino acids (data of strain F32 from Tanzania) [12, 13]. The repetitive sequences of the R2 region of GLURP are highly conserved, but the number of repeat amino acid units (AAUs) varies among *P. falciparum* isolates, resulting in a size polymorphism. Hence, *GLURP* is routinely employed in PCR assays as a genetic tool for parasite identification and studying the multiplicity of malaria infections [34–39]. While the size polymorphism of the *GLURP* R2 region in *P. falciparum* populations is well reported, surprisingly, there is limited information on the sequence variation that underlines the polymorphisms in *GLURP*. To date, most of the available *GLURP* R2 region sequences (and their translated GLURP amino acid sequences) deposited at the NCBI nucleotide database are derived from *P. falciparum* isolates in India [34, 35], with a few unpublished sequences from *P. falciparum* from Iran, Indonesia and China. However, there are no reports of *GLURP* sequences in *P. falciparum* populations in Thailand. To address this deficiency, this study aimed to describe the detailed molecular variation in the *GLURP* R2 region. We generated *GLURP* sequences from 65 parasite isolates from five representative malaria hotspots in Thailand and combined them with the *GLURP* sequence data from *P. falciparum* isolates worldwide, thereby generating a global catalogue of *GLURP* and, after *in silico* translation, GLURP. This collection of *GLURP* sequences can be used to investigate the basis of molecular variations and the signature of natural selection as well as to infer the population structure of *P. falciparum*.

## Methods

### Amplification and DNA sequencing of the *GLURP* R2 region

The DNA templates for the PCR amplification of *GLURP* were extracted from 72 *P. falciparum* samples collected from five localities (Mae Hong Son, Kanchanaburi, Ranong, Trat and Ubon Ratchatani) in Thailand, as previously described [10], and shown in Additional file 1: Figure S1. The species of malaria parasites were confirmed by microscopic examinations of the thin blood film and by genotyping with the genetic markers of merozoite surface protein-1 (*msp*-1) and *msp*-3, as previously reported [40, 41]. For amplification of the R2 region of the *GLURP* gene, the nOF-primer (forward: 5′-CAA CCA AAT CCA CAA GAA CCA GTT GA-3′) and the R2-primer (reverse: 5′-TCT GGT TTA GTG GAT TCA CCT TCA GAT-3′) primer pair were used to yield sequences that corresponded to nucleotide positions 2215–2240 and 3441–3467 of the full-length *GLURP* gene of *P. falciparum* strain F32 (GenBank: M59706.1) [12]. The total PCR reaction volume (50 μl) contained 200 μM of dNTPs, 2 mM of MgCl_2_, 0.3 μM of each primer, 200–300 ng of parasite DNA and 2.5 units of *Taq* polymerase enzyme in 1X *Taq* PCR buffer (Biotechrabbit, Hennigsdorf, Germany). The PCR conditions started with 95 °C for 5 min, followed by 40 cycles of 95 °C for 30 s, 65 °C for 30 s and 72 °C for 90 s, and then followed by a final 72 °C for 5 min. The PCR products were analysed by standard agarose gel electrophoresis in a 2% (*w*/*v*) agarose-TBE gel. The quality of the PCR products was determined under UV transillumination after exposing the gels to ethidium bromide for DNA staining. All the positive PCR products produced a single band and were submitted for commercial automatic DNA sequencing using the Big Dye Terminator System (Applied Biosystems, California, USA) on an ABI3730XL DNA analyser. DNA sequencing was performed twice for each PCR fragment. The sequencing primers included the above PCR primer pair and the additional Int-3 primer (5′-GAA GTG GCT CAT CCA GAA ATT GTT GAA-3′). A total of 65 sequences were unambiguous and included in the present analysis.

### Retrieval of *GLURP* sequences from the NCBI database

A total of 89 nucleotide sequences of the R2 region of the *GLURP* gene from India (*n* = 81), Indonesia (*n* = 3), Iran (*n* = 2) and China (*n* = 1) and the laboratory strains 3D7 and F32 were retrieved from the NCBI nucleotide database (https://www.ncbi.nlm.nih.gov/nuccore) (see Additional file 2: Table S1). In addition, a BLAST_N_ search was performed using the nucleotide sequence of the *P. falciparum* reference strain 3D7 [42] to search against 36 whole-genome shotgun contigs of *P. falciparum* laboratory strains and wild isolates that are deposited in the NCBI database. The whole-genome shotgun contigs with the complete *GLURP* R2 region were identified in the sequences from *P. falciparum* strains HB3, Dd2, FCC-2/Hainan and VS/1, which were submitted by the Broad Institute (Massachusetts, USA), and a further 10 sequences were from *P. falciparum* isolates from Mali (Africa), which were submitted by the Institute for Genome Sciences (Maryland, USA). Taken together, all the *GLURP* R2 region sequences from the 103 *P. falciparum* isolates worldwide and those from the 65 *P. falciparum* Thai isolates in Thailand formed a global database comprised of 168 alleles of the *GLURP* gene.

### Sequence analysis and population genetics analysis

The *GLURP* nucleotide sequences were aligned and then manually adjusted, where necessary, using the BioEdit program [43]. Sequence alignment was performed using ClustalW software [44]. The pairwise interpopulation indices or the Wright *Fst* indices were calculated using the Arlequin software [45]. Other molecular indexes, including the nucleotide diversity (*π*), haplotype index (*hd*), and neutrality tests (Tajima’s *D* test, Fu and Li’s D* test and Fu and Li’s F* test) and linkage disequilibrium, were calculated using the DnaSP software [46, 47].

### Analysis of the AAU composition and GLURP subtypes

Theoretical (*in silico*) translations of the *GLURP* nucleotide sequences were performed using BioEdit software [43], and the resulting GLURP amino acid sequences were exported into a text format. The published amino acid repeat units (AAU) of blocks 1–15 in the R2 region were used to search against the collection of GLURP sequences in order to identify any novel AAU types [34, 35]. A total of 10 AAU types were previously identified between blocks 1–15 of the GLURP R2 region, including one AAU type (*) in block 1, two AAU types (I and II) in block 2, one AAU type (1) in block 3 and 10 AAU types (a, b, c, d, e, f, g, h, i and j) in blocks 4–15 [34, 35]. The arrangements of the AAUs were manually inspected to identify the unique AAU arrangement (GLURP subtype). When novel subtypes were identified, the sequence was BLAST_P_ searched in the NCBI database for confirmation. The total number of AAU types in each block and the parasite isolates with a unique AAU arrangement were recorded. The percentage of AAU types was calculated by dividing the number of a particular AAU type by the total number of AAU types in one block. The diversity of infection (DOI) was calculated by dividing the number of different parasite genotypes that were detected with the total number of parasites that were genotyped [48].

## Results

### Nucleotide diversity and neutrality test

The *GLURP* R2 region was amplified from the genomic DNA of 72 isolates of *P. falciparum* collected from five localities in Thailand. A total of 69 PCR products with single PCR amplicons were submitted for DNA sequencing, generating 65 unambiguous nucleotide sequences. The nucleotide sequences of *GLURP* in *P. falciparum* from Thailand were then combined with 103 sequences from the NCBI nucleotide collections and from whole-genome shotgun sequences to generate a global collection of 168 *GLURP* alleles and the *in silico* translated GLURP alleles in *P. falciparum* isolates worldwide (Additional file 2: Table S1), encoding a total of 105 GLURP subtypes (unique amino acid sequences). The average pairwise nucleotide diversity per site (*π*) and the haplotype index (*hd*) for all 168 *GLURP* samples was 0.00777 ± 0.00066 and 0.757 ± 0.031, respectively (Table 1). The sequence diversity analysis also indicated that the level of genetic diversity in *GLURP* in the *P. falciparum* populations in Thailand was higher than in other populations in Asia but was lower than the parasite population in Africa (Mali) (Table 1). Therefore, these results revealed different levels of sequence diversity in *GLURP* in different demographical locations.

**Table 1:** Summary of statistics for the *GLURP* R2 region in *P. falciparum* populations and tests for departure from neutrality

Population	*n*	GLURP subtype	*hd* ± SD	*π* ± SD	Tajima’s *D*	Fu and Li’s D*	Fu and Li’s F*
Thailand	65	28	0.833 ± 0.038	0.00611 ± 0.00058	-0.53408 (*P* > 0.10)^a^	0.80029 (*P* > 0.10)	0.38828 (*P* > 0.10)
India	81	68	0.944 ± 0.016	0.00377 ± 0.00103	-0.29581 (*P* > 0.10)	-0.88322 (*P* > 0.10)	-0.79358 (*P* > 0.10)
Indonesia	3	3	1 ± 0.272	0.00102 ± 0.00064	nd	nd	nd
Iran	2	1	0	0	nd	nd	nd
China	2	2	1 ± 0.050	0.00159 ± 0.00079	nd	nd	nd
Mali	10	10	1 ± 0.045	0.00915 ± 0.00219	-0.70120 (*P* > 0.10)	-0.73515 (*P* > 0.10)	-0.81915 (*P* > 0.10)
Laboratory lines	5	5	1 ± 0.126	0.01170 ± 0.00439	-0.29817 (*P* > 0.10)	-0.29817 (*P* > 0.10)	-0.31445 (*P* > 0.10)
Global population	168	105^b^	0.757 ± 0.031	0.00777 ± 0.00066	-0.13448 (*P* > 0.10)	-2.05874 (*P* > 0.10)	-0.21350 (*P* > 0.10)

*Abbreviations*: *n* number of sequences, *hd* haplotype diversity index, *π* nucleotide diversity index, *SD* standard deviation, *nd* neutrality tests not determined as the minimum number of sequences required for neutrality tests is four

^a^Statistical significance was deemed at a *P*-value of < 0.05

^b^Total number of GLURP subtypes in the global population is lower than the sum of GLURP subtypes in each population because some GLURP subtypes are detected in more than one population

**Fig. 1:**
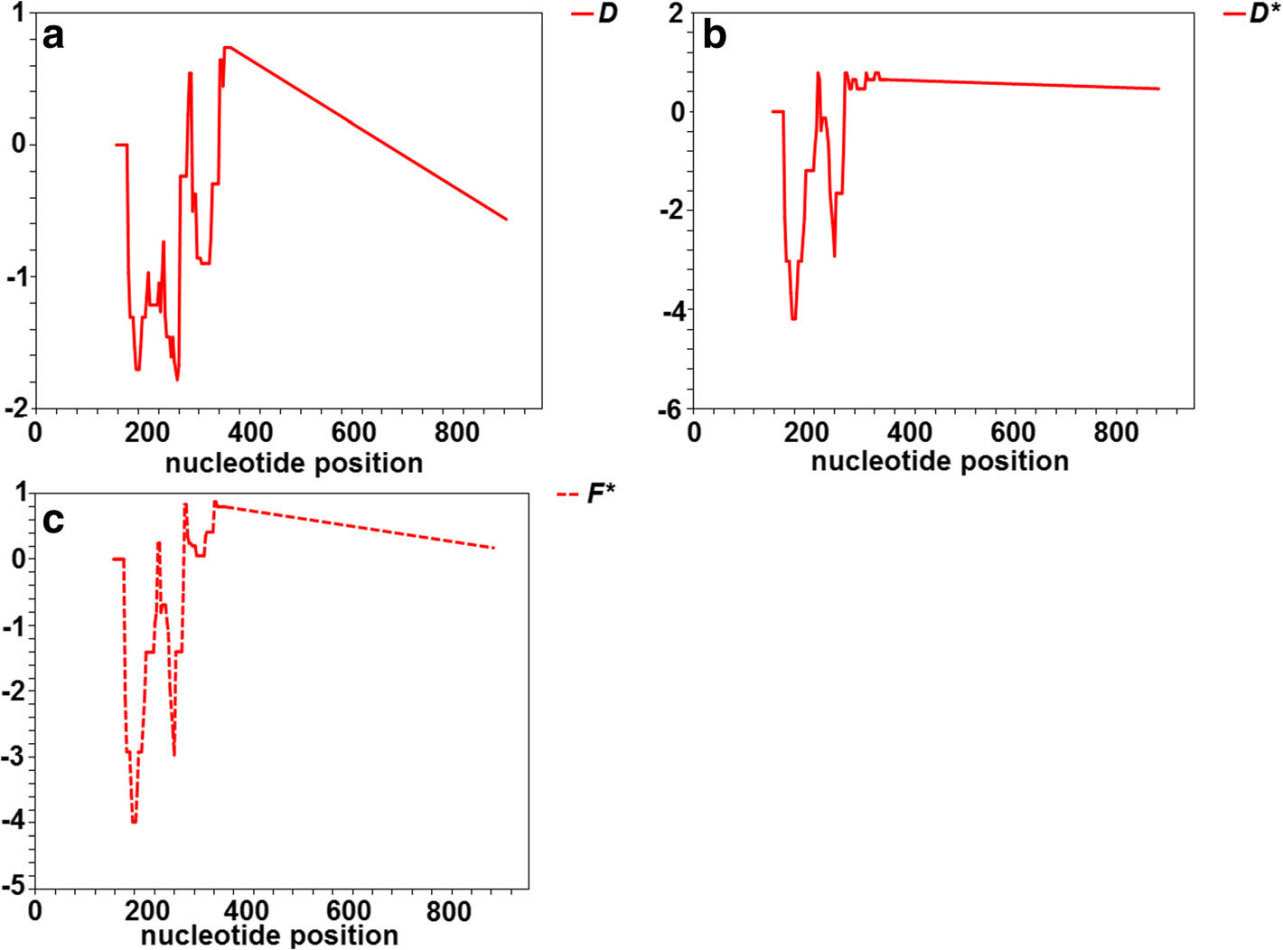
Sliding window plots of the Tajima *D’* (**a**), Fu and Li’s D* (**b**) and Fu and Li F* values (**c**) for the R2 region of the *GLURP* gene of *P. falciparum*. Nucleotide numbers are from the start site of the *GLURP* R2 region. Window length is 25 bp, and the step size is 3 bp. All values were statistically insignificant

Three neutrality tests (Tajima’s *D*, Fu and Li’s D* and Fu and Li’s F* statistics) were performed to examine for any evidence of natural selection in terms of estimating the deviation from neutrality, which is based on the expectation of a constant population size at mutation-drift equilibrium [49, 50]. As shown in Table 1, no departure from neutrality was detected in any of the populations by the three tests. The negative Tajima’s *D* test signified an excess of low frequency polymorphisms relative to the expected level [49]. Similarly, sliding window analyses of the three neutrality tests revealed non-significant values for the *GLURP* R2 region in all three tests (Fig. 1). Therefore, the sequence polymorphisms in the *GLURP* R2 region were apparently neutral, which was likely to be due likely to negative selection.

**Fig. 2:**
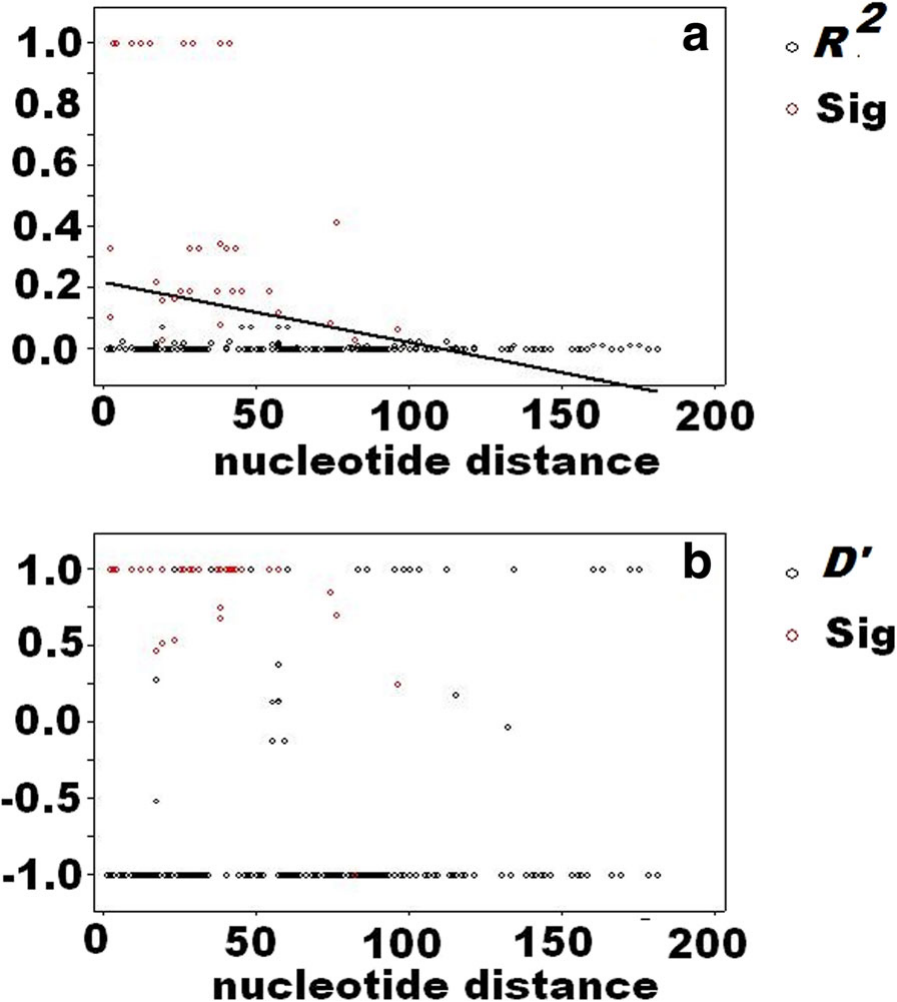
Linkage disequilibrium (LD) across the R2 region of the *GLURP* gene of *P. falciparum* calculated using the *R*^2^(**a**) and *D’* indices (**b**). Red circles represent those pairs of sites that show significant linkage disequilibrium, as calculated by Fisher’s exact test, while all others are shown by black circles. Trace line shows the regression line

Furthermore, when linkage disequilibrium analysis was performed on the *GLURP* R2 region, it revealed a decline in the number of significant linkage disequilibrium between pairs of polymorphic loci (both *R*^2^ and *D’*) over molecular distance (Fig. 2), indicating intragenic recombination in the *GLURP* R2 region. This notion was supported by the presence of five minimum recombination sites, as estimated by means of the four-gamete test (parameter *R*_*m*_). The recombination parameter between adjacent nucleotide sites and for the whole sequence had a value of 0.0129 and 9.2, respectively, which indicates that the meiotic recombination can contribute to the genetic diversity of the *GLURP* gene.

### Composition of the AAUs in the GLURP R2 region

The R2 region of GLURP contained 6–15 blocks of AAUs, which could be classified into 18 types. Sequence alignments of the AAU in each block revealed the conserved polymorphic sites and specific distributions of the 18 AAU types in the R2 region (Additional file 3: Table S2).

Blocks 1 and 3 contained single AAU types (* and 1), which were identical in all the *P. falciparum* isolates. Block 2 was characterized by three AAU types (I, II and III), where type I was the most common type, representing 96% of the block 2 sequence. The AAU types I and II, previously reported in *P. falciparum* populations in India [34, 35], were also detected in the *P. falciparum* populations in Thailand, while AAU type III was novel and was detected in the whole-genome shotgun sequences of *P. falciparum* isolate 309 from Mali (Additional file 2: Table S1). The other 13 AAU types were located between blocks 4–15. Of these, three AAU types (a, b and c), were common in all the *P. falciparum* isolates. In contrast, the other ten AAU types (d, e, f, g, h, i, j, k, l and m) were relatively less common, and their distributions were reserved in specific blocks. Of these, six AAU types (d, e, f, g, h and i) were previously reported in *P. falciparum* populations in India [34, 35], while three others (i, j and l) were detected in the whole-genome shotgun sequences of the *P. falciparum* populations in Mali (Additional file 2: Table S1).

Analysis of the GLURP R2 region in the Thai isolates of *P. falciparum* identified a total of 13 AAU types, including one AAU type in block 1 (*), two AAU types in block 2 (I and II), one AAU type in block 3 (1) and six AAU types (a, b, c, k, l and m) between blocks 4–14. Of these, two AAU types (k and m) were described for the first time in the *P. falciparum* populations in Thailand. Specifically, AAU type k was located in block 5 in the *P. falciparum* isolates RN130 and RN133, while AAU type m was detected in block 12 in the *P. falciparum* isolates RN72 and RN122. These parasite isolates were all from the same population in Ranong Province.

### Size polymorphism in the GLURP R2 region

The GLURP R2 region with a different number of repeat units was detected at different frequencies in the *P. falciparum* populations studied (Table 2). The GLURP with 11 repeat units was the most common GLURP allele, being detected in 53 *P. falciparum* worldwide isolates (31%). In contrast, GLURP with extremely short (6) or long (15) repeat units appeared to be extremely rare. It should also be noted that GLURP with 13 repeat units had the highest allelic diversity, with a total of 26 GLURP subtypes being identified, representing 25% of the total AAU pattern variations (Table 2). In contrast, GLRUP with 6, 8 and 15 repeat units exhibited a lower genetic diversity, with 1, 2 and 2 identified subtypes, respectively.

**Table 2:** Frequency, subtypes and size of the *P. falciparum* GLURP polypeptide in global database. Size indicates the number of amino acid residues detected between blocks 4–15

No. of repeat units	*n*	GLURP subtype	Subtype name	Size (aa residue)
6	1	1	A1	57
8	3	2	C1 to C2	97–98
9	10	4	D1 to D4	115–117
10	27	19	E1 to E19	133–137
11	53	22	F1 to F22	152–156
12	16	14	G1 to G14	172–176
13	37	26	H1 to H26	191–195
14	19	15	I1 to I15	210–214
15	2	2	J1 to J2	229–235

*Note*: The variation in size in blocks 4–15 is due to the insertion/deletion of aspartic acid and glutamic acid between the blocks. The numbers of amino acid residues in blocks 1–3 are invariable (block 1, 20 amino acid residues; block 2, 32 amino acid residues; block 3, 18 amino acid residues). See Additional file 2 for the AAU arrangement of the GLURP subtypes

*Abbreviations*: *aa* amino acid, *n* number of parasites

In addition to the variation in the numbers of repeat units, the size polymorphism of the R2 region of *GLURP* resulted from the insertion or deletion of negatively charged amino acids, including aspartic acid (D) and glutamic acid (E), between the repeat units. The addition of aspartic acid was more frequently detected between blocks 4–9, while the addition of glutamic acid appeared more frequent between blocks 10–12 (Additional file 4: Table S3). Taken together, these results indicated that variations in the repeat units and the insertions/deletions of aspartic acid and glutamic acid between the repeat units contributed to the size polymorphism in GLURP.

### Distribution of the GLURP subtypes and the population structure of *P. falciparum*

The analysis conducted using the 65 alleles of the *GLURP* R2 region from *P. falciparum* populations in Thailand identified 28 GLURP subtypes, containing 8–14 repeat units (Additional file 5: Table S4). The majority (41%) of these Thai *P. falciparum* isolates inherited GLURP with 11 repeat units. This is contradictory to a previous analysis of *GLURP* sequences from Indian populations [34, 35], where the GLURP with 13 repeat units was the most common. The DOI analysis of the Thai *P. falciparum* populations revealed different levels of genetic diversity of GLURP among the Thai subpopulations of *P. falciparum*, where the DOIs of *P. falciparum* at Mae Hong Son, Kanchanaburi and Ranong (Western Thailand) were 0.60, 0.76 and 0.56, while those at Ubon Ratchatani and Trat (Eastern Thailand) were both 0.83. The implication then is that the parasite subpopulations near eastern Thailand were more genetically diverse than those near western Thailand.

Of the Thai GLURP subtypes, the F8 and F11 subtypes were the most common, being identified in 13 and nine *P. falciparum* isolates, respectively, representing 37% of the parasite populations in Thailand (Additional file 5: Table S4). Interestingly, the D3 subtype matched the *GLURP* R2 sequence of *P. falciparum* isolate 2/GL from Indonesia (Eisen et al., unpublished data), while eight other subtypes (D4, E9, F8, F11, H1, H10, H22 and I5) corresponded to the *GLURP* sequences of *P. falciparum* isolates from India [34, 35]. It is also noteworthy that the F11 subtype was identical to the sequence of the *P. falciparum* reference strain Dd2. However, no parasite isolates in Thailand or worldwide had *GLURP* sequences that were similar to those of strains 3D7 and F32, which were employed in the genome sequencing project [42] and the production of the GLURP-based malaria vaccine, respectively [16]. As a result, the present study identified 19 novel GLURP subtypes in Thailand.

The distribution analysis showed that approximately one-third of the GLURP subtypes were shared among the Thai *P. falciparum* subpopulations (Additional file 5: Table S4). To further determine the genetic homogeneity between the parasite populations, pairwise interpopulation comparisons were performed for each parasite population using Wright’s fixation index (*Fst*). The *Fst* values from all pairs of the *P. falciparum* populations were low and non-significant (Table 3), indicating a similar distribution of the *GLURP* subtypes in the five *P. falciparum* populations in Thailand. Thus, gene flow might have operated in *P. falciparum* subpopulations from the Thailand-Myanmar, Thailand-Laos and Thailand-Cambodia borders. In addition, the *Fst* index for the *GLURP* alleles from *P. falciparum* in Thailand and India (*n* = 81) was -0.00312 (*P =* 0.24). This low and non-significant *Fst* value indicated that there was genetic homogeneity in the GLURP subtypes between these *P. falciparum* populations in Thailand and India.

**Table 3:** Pairwise *F*_*st*_ indices of the *GLURP* alleles between *P. falciparum* populations in Thailand. *Fst* values were low and non-significant in all pairs of *P. falciparum* populations

	K	MH	RN	TD
MH	-0.00499	–		
	(*P* = 0.40)			
RN	0.02430	-0.58080	–	
	(*P* = 0.23)	(*P* = 0.81)		
TD	-0.04670	-0.06146	0.00448	–
	(*P* = 0.80)	(*P* = 0.68)	(*P* = 0.32)	
UB	0.07521	0.05245	0.07187	0.07195
	(*P* = 0.11)	(*P* = 0.16)	(*P* = 0.10)	(*P* = 0.14)

*Abbreviations*: *MH* Mae Hong Son, *K* Kanchanaburi, *RN* Ranong, *TD* Trat, *UB* Ubon Ratchatani

## Discussion

The present study reports the genetic structure and diversity of the immunodominant GLURP R2 region in *P. falciparum* populations in Thailand and worldwide. As of September 2017, 103 sequences of the *GLURP* gene were available in the NCBI database. Sequence analysis led to the identification of 86 unique GLURP subtypes. In the present study, 65 *GLURP* sequences were generated from *P. falciparum* populations in Thailand, and 28 GLURP subtypes were identified, including 19 new subtypes that were identified for the first time. Overall, the combined database of GLURP revealed 105 GLURP subtypes in 168 alleles of the *P. falciparum* isolates worldwide.

Variations in the repeat unit numbers led to size polymorphisms in the GLURP R2 region. In the present study, GLURP, with 8–14 repeat unit blocks was detected in *P. falciparum* in Thailand, with 11 repeat unit blocks being the most frequent. This result contrasts with previous analyses of GLURP sequences from *P. falciparum* in Indian isolates [34, 35], where *GLURP* genes with 9–15 repeat unit blocks were detected, with 13 repeat units being the most common. Within the whole-genome shotgun sequences of *P. falciparum* laboratory strains, the shortest *GLURP* R2 region was found to contain only six repeat units, which was detected in *P. falciparum* HB3. Overall, the data demonstrated the extensive size variation in the *GLURP* gene in both laboratory strains and natural isolates of *P. falciparum*.

Comparison of the amino acid repeat units revealed conserved amino acid substitutions amongst the repeat units. Of the 18 AAU types identified, the majority were located between blocks 4–15, with fewer AAU types in blocks 1–3. Indeed, blocks 1 and 3 had identical sequences in all *P. falciparum* isolates. The limited amino acid substitutions in the AAU likely reflects the functional constraints of the GLURP protein. A recent study reported that GLURP formed the Pfs38 protein complex on the merozoite surface that binds to erythrocytes via glycophorin A and participates in the parasite invasion of erythrocytes [14]. This is consistent with the three neutrality tests (Tajima’s *D*, Fu and Li’s D* and Fu and Li’s F* tests) that all gave non-significant values, suggesting that the conserved AAU sequences were maintained under negative purifying selection. Together, these results indicated that the AAU sequences of GLURP are highly conserved, supporting the view that GLURP is an attractive target for vaccine development [17–21].

Another important finding in the present study was that the *GLURP* R2 region in Thai populations of *P. falciparum* exhibited extensive sequence variation, with a total of 13 AAU types and 28 GLURP subtypes. The majority of these GLURP subtypes (19 subtypes, 67%) in Thailand were novel. However, the number of GLURP subtypes in *P. falciparum* in Thailand was lower than that in Indian isolates, where 61 GLURP subtypes were seen [34, 35]. This contrasts to a previous genetic analysis of *P. falciparum* using *msp-3* gene sequences, in which fewer *msp-3* haplotypes were detected in the *P. falciparum* populations in India than in Thailand [41]. Accordingly, the genetic diversity in *P. falciparum* populations should be evaluated using multiple genetic loci. If genetic markers that exhibit low or limited polymorphisms are employed for genotyping, it is likely that the genetic variation of parasite populations will be underestimated. Because GLURP is highly polymorphic in size and is not under the influence of positive selection, this marker would be suitable for epidemiological and population genetic studies.

The mechanisms of the molecular variation in *GLURP* can be summarized as follows. First, the emergence of the novel GLURP subtypes was attributed to non-synonymous amino acid substitutions in different repeat units that resulted in the formation of new AAU types. Of the 18 AAU types identified in *P. falciparum* worldwide, two AAU types (l and m, see Additional file 3: Table S2) are reported here for the first time. In addition, novel GLURP subtypes might have arisen from genetic recombination, resulting in a new assortment of AAU types, as well as by the insertion and deletion of extra amino acids, including aspartic acid (D) and glutamic acid (E), between the repeat units. Taken together, our study revealed the mechanisms contributing to the genetic diversity of the *GLURP* gene in *P. falciparum* populations.

Comparison of GLURP subtypes within the Thai *P. falciparum* populations revealed different levels of genetic diversity among the different *P. falciparum* populations. From the DOI indices, *P. falciparum* populations in eastern Thailand were more diverse than those in western Thailand. The low DOI simply reflected that the *P. falciparum* populations in Mae Hong Son, Kanchanaburi and Ranong had large numbers of individuals with the same GLURP subtypes. The analysis also showed that different subpopulations of *P. falciparum* in Thailand shared a large number of GLURP subtypes, indicating that gene exchange may occur between the parasites near the eastern and western borders of Thailand. This hypothesis was supported by the Wright’s statistical analysis, where low and non-significant *Fst* values were found for all the pairs of compared *P. falciparum* populations. In addition, the *Fst* analysis of the GLURP subtypes in *P. falciparum* in India and Thailand also showed a low and non-significant *Fst* value, indicating similar GLURP alleles between the parasite populations. Together, these results demonstrated that *P. falciparum* populations in different geographical locations may exhibit different levels of genetic diversity, but gene flow likely exists among the *P. falciparum* populations in the Greater Mekong Subregion and India.

The sequence analysis and population structure presented here may be useful for developing future GLURP-based vaccines and for monitoring vaccine efficacy. Currently, the GMZ2 vaccine, which incorporates the GLURP R0 region of *P. falciparum* strain F32 and MSP-3, is being tested in clinical studies in various endemic areas [32, 51]. Although GMZ2 reduced the malaria incidence, its efficacy was still low and so vaccines using a more immunogenic formulation are needed. One proposed strategy is to incorporate the GLURP R2 region of *P. falciparum* strain Dd2 in a vaccine, since the R2 region also contains B-cell epitopes and might elicit functional protective antibodies [52], while the most common GLURP subtype (F11) in Thailand and India is identical to that of Dd2. Thus, such a vaccine might exhibit improved efficacy against clinical cases of malaria.

## Conclusions

The present study generated a database of *GLURP* gene sequences, and the *in silico* translated GLURP sequences, from worldwide populations of *P. falciparum*. The database revealed not only the sequence diversity but also the distribution of the GLURP subtypes. Because the *GLURP* gene exhibited extensive DNA size and sequence polymorphism, it might serve as a genetic marker for parasite identification as well as for population and epidemiological studies. The sequence information provides knowledge for a better design of GLURP-based malaria vaccines and reveals the population structure of *P. falciparum* across Thailand, which is of use for monitoring and controlling this parasite.

## Additional files

Additional file 1: Figure S1. Map showing the sampling site locations for *Plasmodium falciparum* collection in Thailand. *Abbreviations*: MH, Mae Hong Son; K, Kanchanaburi, RN, Ranong; UB, Ubon Ratchatani; TD, Trat. (DOC 67 kb)

Additional file 2: Table S1. The AAU arrangement in 168 alleles of GLURP in *P. falciparum* isolates worldwide. The capital letters D and E represents the additions of aspartic acid and glutamic acid, respectively, between the repetitive regions (blocks 4–14). The lowercase letters in blocks 4–14 indicate the amino acid repeat unit (see also Additional file 3: Table S2). (XLS 133 kb)

Additional file 3: Table S2. Amino acid sequences and frequency (in %) of AAUs in the R2 region of the GLURP gene in *P. falciparum*. AAUs were named according to the GLURP sequences of *P. falciparum* in India [34, 35]. Bold letters indicate the amino acid substitutions that define AAU type (XLS 43 kb)

Additional file 4: Table S3. The percentage of the aspartic acid (D) and glutamic acid (E) insertions at the end of Block 4 to 15 sequences in R2 region of *P. falciparum GLURP* gene. (DOC 34 kb)

Additional file 5: Table S4. Distribution of the GLURP subtypes in 65 *P. falciparum* isolates in Thailand. Bold letters indicate the 10 GLURP subtypes that are prevalent in more than one endemic site in Thailand (DOC 92 kb)
