# Supplementary material for: Size and sequence polymorphisms in the glutamate-rich protein gene of the human malaria parasite Plasmodium falciparum in Thailand
Source: Parasit Vectors. 2018 Jan 22;11:49. doi: 10.1186/s13071-018-2630-1 (PMC5778735; doi:10.1186/s13071-018-2630-1)
Supplement: Supplementary file 1 — Map showing the sampling site locations for Plasmodium falciparum collection in Thailand. Abbreviations: MH, Mae Hong Son; K, Kanchanaburi, RN, Ranong; UB, Ubon Ratchatani; TD, Trat. (DOC 67 kb) [file 13071_2018_2630_MOESM1_ESM.doc]

**Additional file 1**

**Figure S1**


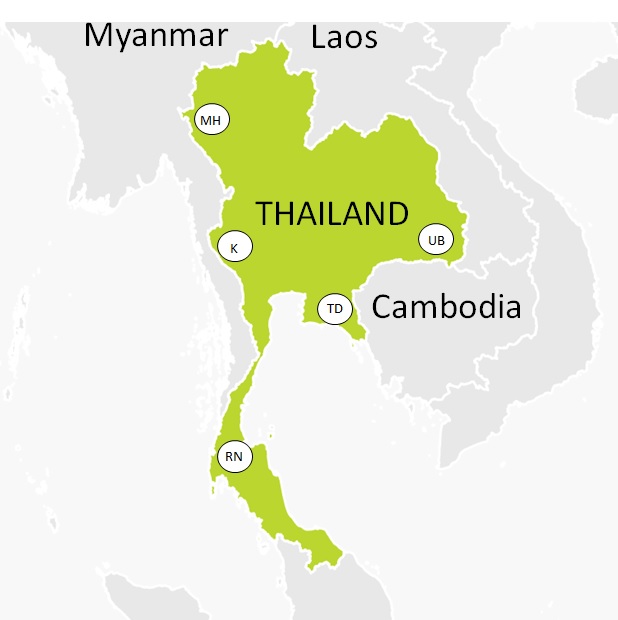
A map of *Plasmodium falciparum* sampling sites in Thailand. MH, Mae Hong Son; K, Kanchanaburi; RN, Ranong; TD, Trat; UB, Ubon Ratchatani.
