# Supplementary material for: Size and sequence polymorphisms in the glutamate-rich protein gene of the human malaria parasite Plasmodium falciparum in Thailand
Source: Parasit Vectors. 2018 Jan 22;11:49. doi: 10.1186/s13071-018-2630-1 (PMC5778735; doi:10.1186/s13071-018-2630-1)
Supplement: Supplementary file 4 — The percentage of the aspartic acid (D) and glutamic acid (E) insertions at the end of Block 4 to 15 sequences in R2 region of P. falciparum GLURP gene. (DOC 34 kb) [file 13071_2018_2630_MOESM4_ESM.doc]

**Additional file 4**

|  | *n* | D | E | D+E |
| --- | --- | --- | --- | --- |
| Block 4 | 168 | 5% | 0% | 5% |
| Block 5 | 168 | 14% | 1% | 15% |
| Block 6 | 168 | 25% | 0% | 25% |
| Block 7 | 167 | 23% | 2% | 25% |
| Block 8 | 167 | 12% | 4% | 16% |
| Block 9 | 164 | 30% | 15% | 45% |
| Block 10 | 154 | 14% | 18% | 32% |
| Block 11 | 127 | 9% | 10% | 19% |
| Block 12 | 82 | 13% | 20% | 33% |
| Block 13 | 56 | 11% | 7% | 18% |
| Block 14 | 20 | 5% | 5% | 10% |
| Block 15 | 2 | 0% | 0% | 0% |

**Table S3** The percentage of the aspartic acid (D) and glutamic acid (E) insertions at the end of Block 4 to 15 sequences in R2 region of *P. falciparum* *GLURP* gene.
