# Supplementary material for: Size and sequence polymorphisms in the glutamate-rich protein gene of the human malaria parasite Plasmodium falciparum in Thailand
Source: Parasit Vectors. 2018 Jan 22;11:49. doi: 10.1186/s13071-018-2630-1 (PMC5778735; doi:10.1186/s13071-018-2630-1)
Supplement: Supplementary file 5 — Distribution of the GLURP subtypes in 65 P. falciparum isolates in Thailand. Bold letters indicate the 10 GLURP subtypes that are prevalent in more than one endemic site in Thailand (DOC 92 kb) [file 13071_2018_2630_MOESM5_ESM.doc]

**Additional file 5**

**Table S4** Distribution of the GLURP subtypes in 65 *P. falciparum* isolates in Thailand. Bold letters indicate the 10 GLURP subtypes that are prevalent in more than one endemic site in Thailand

| **Repeat unit** | **GLURP subtype** | ***n*** | **Origin** | | | | | **Parasite with the same allele of *GLRUP*** | **Origin of the reference strain** | **Reference** |
| --- | --- | --- | --- | --- | --- | --- | --- | --- | --- | --- |
|  |  |  | **MH (DOI = 0.60)** | **K (DOI = 0.76)** | **RN (DOI = 0.56)** | **TD (DOI = 0.83)** | **UB (DOI = 0.83)** |  |  |  |
| 8 | **C1** | 2 | – | K392 | – | – | UB82 | – |  |  |
| 9 | **D3*** | 2 | – | K185 | RC17 | – | – | 2/GL | Indonesia | Eisen et al., unpublished data |
|  | **D4*** | 3 | – | K205 | – | – | UB27, | JKQ2084 | India | [34] |
|  |  |  |  |  |  |  | UB28 |  |  |  |
| 10 | E1 | 1 | MH11 | – | – | – | – | – |  |  |
|  | E3 | 1 | – | – | RN68 | – | – | – |  |  |
|  | E4 | 1 | – | – | – | TD556 |  | – |  |  |
|  | E9* | 1 | – | K402 | – | – | – | 2011B, | India | [35] |
|  |  |  |  |  |  |  |  | 2011C, | India |  |
|  |  |  |  |  |  |  |  | 2011D, | India |  |
|  |  |  |  |  |  |  |  | 2011J | India |  |
|  | E10 | 1 | – | K386 | – | – | – | – |  |  |
|  | E19 | 2 | – | – | – | TD504, | – | – |  |  |
|  |  |  |  |  |  | TD508 |  |  |  |  |
| 11 | F1 | 1 | – | – | RN131 | – | – | – |  |  |
|  | **F8*** | 13 | MH50, | K66, | RN28, | TD531, | – | 005C, | India |  |
|  |  |  | MH51, | K215 | RN63, | TD533, |  | 005E |  | [35] |
|  |  |  | MH61, |  | RN70 | TD535, |  |  |  |  |
|  |  |  | MH66 |  |  | TD554 |  |  |  |  |
|  | **F11*** | 9 | MH18, | K58, | RN19, | – | UB14 | 005G, | India |  |
|  |  |  | MH20 | K165, | RN31, |  |  | 005P, | India | [34, 35] |
|  |  |  |  | K397 | RN36 |  |  | 2005O, | India |  |
|  |  |  |  |  |  |  |  | 2011K, | India |  |
|  |  |  |  |  |  |  |  | JKQ675, | India |  |
|  |  |  |  |  |  |  |  | Dd2 | Laos (Indochina) |  |
|  | F14 | 1 | – | K1 | – | – | – | – |  |  |
|  | **F15** | 2 | – | – | RN129 | – | UB85 | – |  |  |
|  | F21 | 1 | – | K403 | – | – | – | – |  |  |
| 12 | G1 | 1 | – | K60 | – | – | – | – |  |  |
|  | G12 | 2 | – | K74, | – | – | – | – |  |  |
|  |  |  |  | K391 |  |  |  |  |  |  |
|  | G14 | 2 | – | - | RN130, | – | – | – |  |  |
|  |  |  |  |  | RN133 |  |  |  |  |  |
| 13 | H1* | 1 | – | – | – | – | UB58 | JKQ6387 | India | [34] |
|  | H3 | 1 | – | – | – | – | UB51 | – |  |  |
|  | **H7** | 2 | MH32 | – | – | – | UB59 | – |  |  |
|  | **H10** | 2 | – | - | – | TD510 | UB50 | – |  |  |
|  | H10* | 1 | – | K195 | – | – | – | JKQ841 | India | [34] |
|  | **H23*** | 5 | MH10 | - | RN26, | TD529 | UB84 | JKQ1912, | India | [34, 35] |
|  |  |  |  |  | RN66 |  |  | 2011H | India |  |
|  | H24 | 2 | – | - | RN72, | – | – | – |  |  |
|  |  |  |  |  | RN122 |  |  |  |  |  |
| 14 | I5* | 2 | – | – | – | – | UB7, | 2011F | India | [35] |
|  |  |  |  |  |  |  | UB52 |  |  |  |
|  | **I8** | 2 | MH24 | – | – | TD530 | – | – |  |  |
|  | I11 | 1 | – | K389 | – | – | – | – |  |  |

*Abbreviations*: DOI, diversity of infection; MH, Mae Hong Son; K, Kanchanaburi; RN, Ranong; TD, Trat; UB, Ubon Ratchatani

*Asterisks indicate nine GLURP subtypes of *P. falciparum* in Thailand that match the published sequences in the NCBI database
